# Understanding peri‐implantitis occurrence and recurrence following treatment: A patient‐centered study

**DOI:** 10.1002/jper.70133

**Published:** 2026-04-28

**Authors:** Alberto Monje, Manal Sarsri Reffassi, Cristina Vallés, Ilyes Gourdache, Purnima Kumar, José Nart

**Affiliations:** ^1^ Department of Periodontology Universitat Internacional de Catalunya Barcelona Spain; ^2^ Department of Periodontology and Oral Medicine University of Michigan Ann Arbor Michigan USA

**Keywords:** complication endosseous implant, dental implant, mucositis, peri‐implant disease, peri‐implantitis

## Abstract

**Background:**

The present study aimed at gaining insight on patients´ perception of peri‐implantitis and its treatment and also to explore the effect of treatment outcome on patients´ perception and satisfaction.

**Methods:**

Patients who had received peri‐implantitis treatment were contacted and invited to participate in the questionnaire‐based survey. Overall, 30 items were surveyed, including demographics, data concerning implant placement, diagnosis and signs/symptoms of peri‐implantitis, level of education of disease, risk factors, knowledge of factors implicated in disease recurrence, and treatment delivered along with its self‐perceived outcomes. Moreover, the treatment outcome was further recorded. Descriptive analyses and correlation tests were applied to explore association.

**Results:**

Based on an a priori sample size calculation, 100 patients with 258 implants subjected to peri‐implantitis treatment were included. In total, 63% of the patients successfully responded to therapy displaying arrest in progressive bone loss, 26% demonstrated progressive bone loss (≥ 1 mm) and 11% were subjected to implant removal. The overall level of knowledge about peri‐implantitis was moderately low, where ∼ 60% of the patients surveyed reported null knowledge about diagnosis, prevalence, or risk factors associated with disease occurrence, despite having received treatment of this disease. Compliers with supportive care demonstrated greater level of concern and knowledge regarding peri‐implantitis and its recurrence. Patients were overall satisfied immediately (following therapy), at early (re‐evaluation), and at late stages (during follow‐up) with therapeutic outcomes of peri‐implantitis, despite the therapeutic outcome achieved. In addition, patients accepted the possibility of disease recurrence and their role in minimizing it.

**Conclusion:**

Patient‐reported information on factors related to peri‐implantitis occurrence and recurrence is suboptimal. Nearly 60% of the patients are unaware of factors related to peri‐implantitis ocurrence. However, peri‐implantitis therapy does not seem to negatively interfere in the patients´ quality of life or satisfaction, despite the treatment outcomes.

**Plain language summary:**

This study examined how patients understand peri‐implantitis and how they feel about its treatment. One hundred patients who received peri‐implantitis therapy completed a questionnaire, and treatment outcomes were evaluated using x‐rays. Most patients had limited knowledge about peri‐implantitis, its causes, and risk factors, even after treatment. Patients who attended regular follow‐up care showed greater awareness of the disease. Despite differences in treatment outcomes, including ongoing bone loss or implant removal, patients were generally satisfied with their treatment and accepted the possibility of disease recurrence. These findings highlight the need for better patient education, while showing that peri‐implantitis treatment does not negatively affect patient satisfaction.

## 1 INTRODUCTION

The longevity of dental implants can be compromised by the occurrence of biological complications. Peri‐implantitis has been regarded as the primary reason for implant failure.^1^ Peri‐implantitis is characterized by tissue breakdown.^2^ The disorder is initiated with an inflammation confined within the soft tissues (peri‐implant mucositis) which, if not treated, can be the precursor to peri‐implantitis. Peri‐implantitis affects about two in 10 implants, and nearly 1 in 5 patients.^3^ Several factors have been identified that increase the risk for disease either by altering the peri‐implant microbiome to a dysbiotic state, or by creating florid, unresolved inflammation, or both. These factors are both at the level of the patient (e.g., smoking, diabetes) and at the level of the implant (implant position, emergence profile, and angle of the restoration, etc.)^4^

With the growing concern of peri‐implantitis, patients´ perspectives and perceptions of implant longevity and complications have been recently explored, and inaccurate perceptions and unrealistic expectations of implant therapy have been reported.^5^, ^6^ Many patients are unaware of disease, even when symptoms occur.^7^ In fact, ∼ 70% of patients believed that peri‐implantitis is a rare disease and that implants are life‐lasting devices.^8^ Even among patients with both periodontitis and peri‐implantitis, the levels of disease perception were low, and most patients underestimated the importance of their role in disease control.^9^ These perceptions, along with ignorance regarding the factors that contribute to peri‐implantitis^8^ serve to increase anxiety when a disease diagnosis is conveyed.^10^ By contrast, the diagnosis of peri‐implant disease showed significant correlation with the quantity and quality of information provided at the time of placement.^11^

Non‐surgical and surgical measures to manage peri‐implantitis have demonstrated to be associated with significant post‐surgical recession.^12^, ^13^, ^14^ It is speculated that this may lead to suboptimal patient‐reported outcomes. However, there is a paucity of data concerning the perspective of patients concerning the therapy of peri‐implantitis and its treatment outcomes and complications. To the authors´ best knowledge, only one study assessed the impact of surgical therapy on patients´ quality of time in the short‐term.^15^ Interestingly, it was demonstrated that postoperative pain was mild to moderate and that the treatment of peri‐implantitis has an impact on quality of life, especially when augmentation procedures were involved.^15^ However, the short‐term study period and the exclusion to non‐surgical therapeutic modalities do not allow to draw robust conclusions.

Therefore, the present study aimed at gaining insights into patients´ perception of peri‐implantitis and its treatment to enhance communication strategies prior to therapy to avoid false expectations and to explore the effect of treatment outcomes on patients´ perception of the treatment and their satisfaction. As secondary objective, this study aimed at patients’ understanding of the harm and benefits concerning the treatment of peri‐implantitis. The hypotheses of this research project are that patients´ knowledge, including etiology and risk factors, of peri‐implantitis is limited and that peri‐implantitis is associated with esthetic (based upon any patient‐reported mucosal deformity) and phonetic sequela that alter patients´ satisfaction and wellness. Moreover, it was hypothesized that treatment outcomes may impact patients´ perception and satisfaction.

## 2 MATERIALS AND METHODS

This study was reported according to the STROBE guidelines. The present study was designed as a population‐based cross‐sectional survey of patients attending the Department of Periodontology at Universitat Internacional de Catalunya (ethics committee: PER‐ECL‐2024‐08—Barcelona, Spain). The registration of this clinical study was not required. The study was performed following the approval of the Ethics Committee and was conducted according to the principles outlined in the Declaration of Helsinki and Ethical Conduct for Research with Human Beings revised in 2013. All participants gave their informed consent prior to their inclusion in the study.

The survey was developed to gather insights into patient‐related outcomes regarding implant placement, diagnosis, and treatment of peri‐implantitis. Its design was based on previously reported questionnaires.^10^, ^16^ In summary, 30 items were surveyed, including demographics, data concerning implant placement, the diagnosis and signs/symptoms of peri‐implantitis, level of disease literacy, risk factors, recurrence, and treatment delivered along with its perceived outcomes. The survey instrument is shown in Supplementary Table 1.

In addition, periapical X‐rays were collected retrospectively at baseline (during diagnosis of peri‐implantitis) and at latest follow‐up (at the time the patients completed the survey) to assess whether progressive bone loss occurred or not following delivery of the therapy. Clinical parameters were not collected due to the heterogeneity among examiners and the different indices used over the study period.

### 2.1 Sample size calculation

A sample size of 100 patients was deemed necessary and sufficient to provide a maximum error of 10% to estimate a proportion over the total population (assuming 95% of confidence and *p* = 0.5 for that proportion). Sample size was calculated according to the main outcome variable (i.e., patients’ perspective of peri‐implantitis and its treatment). In addition, 88.2% of statistical power was reached for detecting a medium‐large effect size (*d* = 0.65) in differences of distributions between different categories as significant considering 95% confidence.

### 2.2 Recruitment

Patients attending to the Department of Periodontics at Universitat Internacional de Catalunya (UIC) Barcelona (Barcelona, Spain) were screened for eligibility. Moreover, e‐mail invitations were sent to patients that underwent to peri‐implantitis treatment according to the records. Adult patients ≥ 18 years old with at least one dental implant diagnosed of peri‐implantitis and that received therapy ≥ 3 months ago were included. The diagnosis of peri‐implantitis was carried out according to the Consensus Report of Workgroup 4 of the 2017 World Workshop on the Classification of Periodontal and Peri‐Implant Diseases and Conditions.^17^ Patients with self‐reported neurological, cognitive, or psychiatric conditions and diseases, or other known conditions to influence their ability to objectively answer the questionnaire were also excluded. Accordingly, one‐hundred patients were estimated and were invited to complete one questionnaire based on their experience in the diagnosis and treatment of peri‐implantitis.

### 2.3 Questionnaire 1: Diagnosis

The first questions focused on the patients’ demographic and clinical background, including gender, age, number of dental implants, follow‐up since installation, and whether they were placed at the university (and the department) or elsewhere. Patients were also surveyed about their compliance with regular recall and monitoring, specifying the frequency of these appointments. Subsequent questions aimed at exploring the diagnosis of peri‐implantitis, asking patients when the infection was diagnosed, whether they noticed any initial symptoms (such as bleeding, pain, swelling, or bad breath), and who first detected the condition (dentist, periodontist, dental hygienist, or other professional). The number of infected implants diagnosed with peri‐implantitis was also recorded.

### 2.4 Questionnaire 2: Knowledge and perception

The next group of questions addressed patients’ knowledge and perception of peri‐implantitis, using 5‐point scale to assess how well they understood its diagnosis, risk factors, preventive elements, and current prevalence. The following section aimed at evaluating the perceived risk of various contributing factors, including poor oral hygiene, smoking, diabetes, previous periodontitis, and non‐compliance with regular maintenance, as well as clinical and technical elements such as implant surface, occlusion, and prosthesis type.

### 2.5 Questionnaire 3: Therapeutic outcome

The items examined treatment characteristics and related outcomes. Patients were surveyed whether they received surgical or non‐surgical therapy, and if specific interventions were used (bone or soft tissue grafts, membranes, laser), which were later verified from the clinical records. Questions also related to post‐treatment care, including the use of antibiotics, painkillers, or mouth rinses; the survival of their treated implants; and the information they received regarding the expected success rate of their treatment. Patients were asked to rate their level of concern about post‐treatment pain, esthetics, inflammation, or infection both immediately after treatment and following re‐evaluation. Finally, patients rated their overall satisfaction and perception toward implant therapy and peri‐implantitis management, indicating how likely they were to recommend or repeat the treatment, whether they believed the problem had been solved, and how much they agreed with statements about disease seriousness, recurrence, personal responsibility, and the role of the surgeon.

### 2.6 Therapeutic outcome

Periapical radiographs from included implants were retrospectively retrieved to explore whether therapy succeeded in arresting progressive bone loss. Accordingly, implants were categorized as arrested bone loss (≥ 1 mm)^18^ for implants that did not show progressive bone loss at latest follow‐up when compared to baseline examination (previous to therapy), progressive bone loss (≥ 1 mm) of implants subjected to therapy or implants that were opted to be removed.

### 2.7 Statistical analysis

A general descriptive analysis was performed for categorical variables using absolute and relative frequencies and mean, standard deviation, range, median, and interquartile range (IQR) for the continuous scores. Chi‐squared^2^ test was applied to assess the association or dependence between categorical variables. Mann–Whitney´s tests were used to compare distributions of ordinal‐scale questions among two independent groups. Pearson´s correlation coefficients were calculated to measure the degree of association between ordinal‐scale variables with the different demographic features evaluated. Moreover, Pearson´s correlation was also applied to test the correlation between level of satisfaction and treatment outcomes. Significance level used in analysis was set at 5*%* (*α* = 0.05).

## 3 RESULTS

**FIGURE 1:**
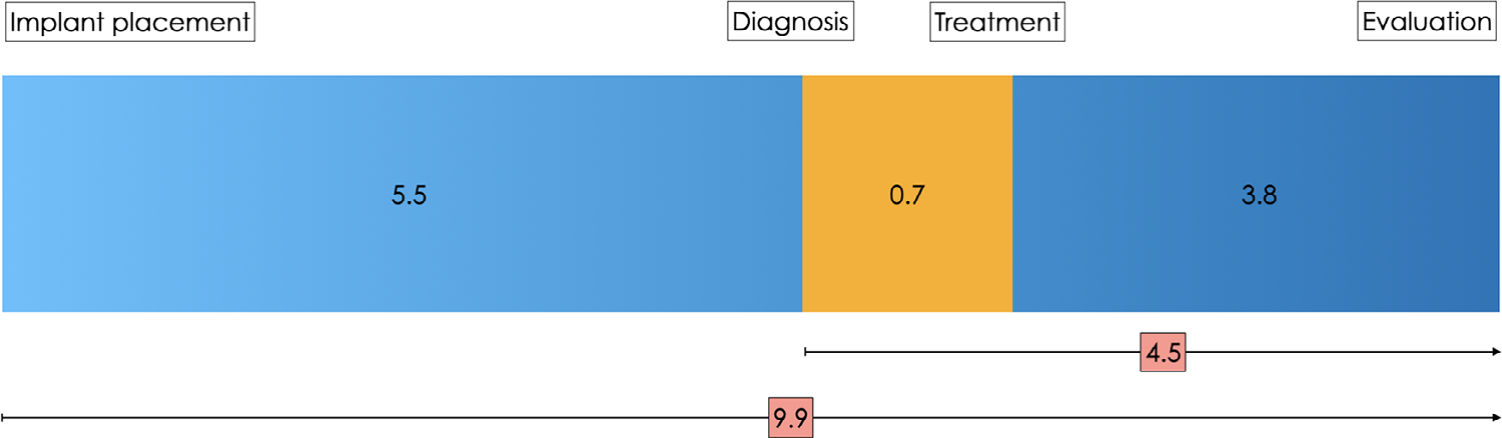
Timeframes (years) since implant placement, diagnosis, treatment and evaluation.

Overall, 140 patients were screened. Of these, two were excluded due to their unwillingness to participate in the study, and 38 were excluded due to inability to properly address the questionnaire due to cognitive/motor disorders. In total, 44% were males and 56% females. Mean age was 63.6 ± 10.7 years. The total number of implants within the included patients was 515 with a mean number of implants being 5.2 ± 3.4 (range 1–14). Mean time since implant placement was 9.7 ± 5.2 years (range 1–25). In total, 258 implants treated due to peri‐implantitis were included. In mean, 4.5 ± 3.1 years were the treated implants following diagnosis of peri‐implantitis (Figure 1). Non‐surgical mechanical debridement was provided in 76% of the individuals, surgical therapy on 16% and both in 8%. Patients where non‐surgical therapy succeeded or declined due to financial reasons, maintenance was prescribed. Antibiotics were prescribed in 45% of the patients, 63% reported using mouth rinse and 36% pain killers for 5 + days. Two thirds of patients (67%) were compliers, attending regularly the supportive periodontal/peri‐implant care (SPC) sessions to recall and maintenance visits (usually twice a year: 83.1%).

### 3.1 Knowledge of diagnosis and risk factors

**FIGURE 2:**
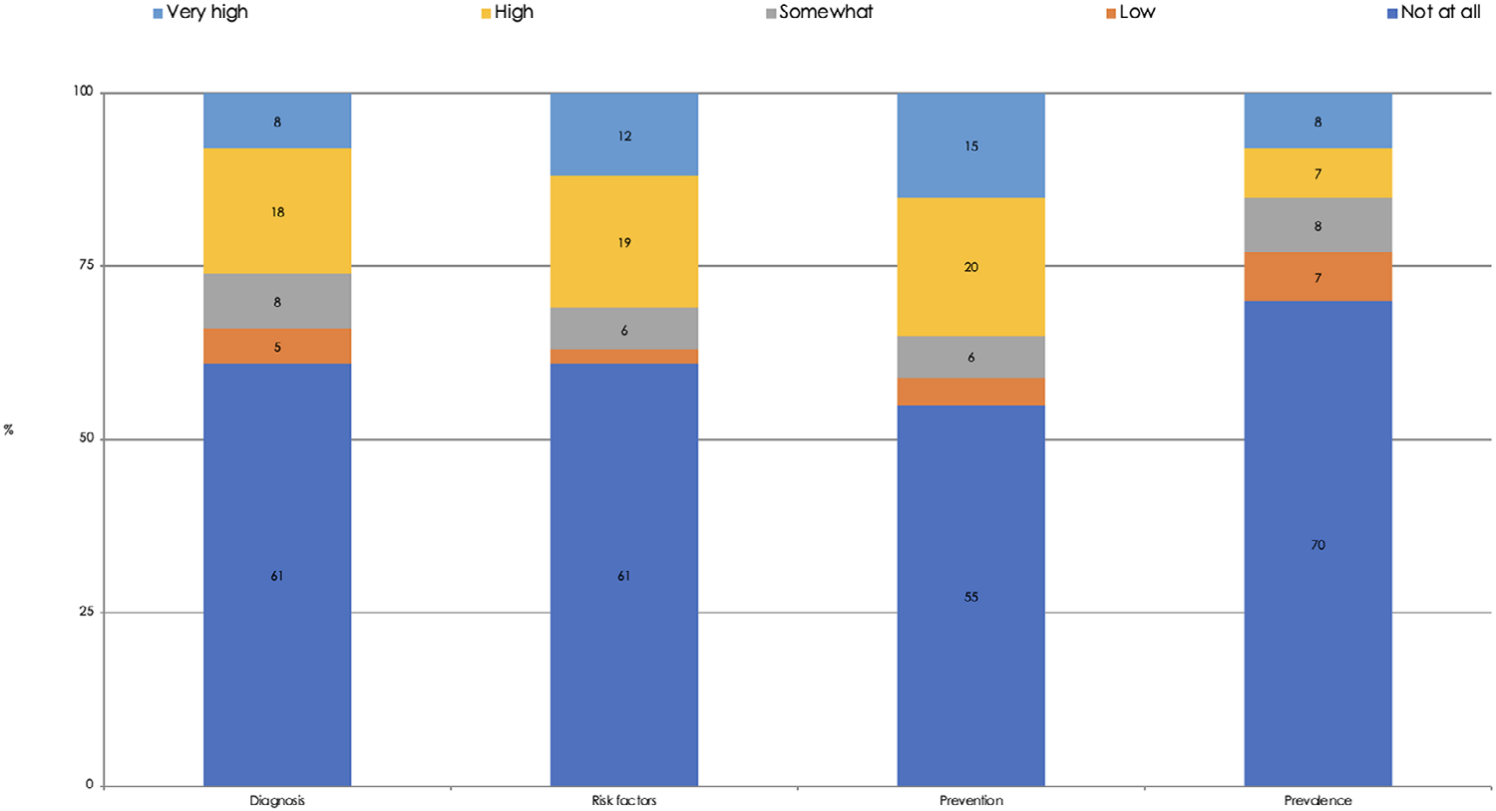
Overall knowledge about peri‐implantitis in patients subjected to treatment.

**FIGURE 3:**
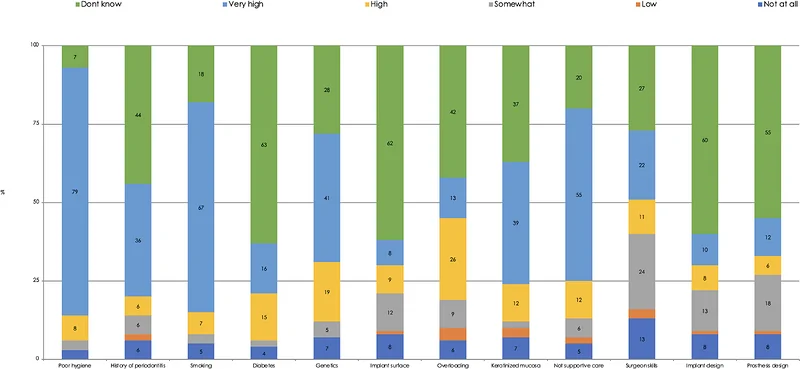
Overall knowledge about factors associated with peri‐implantitis in patients subjected to treatment.

The vast majority (83%) of patients were not alarmed by symptoms, with pain (35.3%) and profuse bleeding upon brushing (23.5%) being the most commonly reported. The periodontist was most commonly the specialist who provided the diagnosis of peri‐implantitis. Level of ignorance related to diagnosis, risk factors, preventive elements, and prevalence of peri‐implantitis was significant (55%–70%, Figure 2). Age and level of compliance with supportive care demonstrated significant associations with knowledge levels. Older individuals tended to demonstrate lower knowledge of diagnosis (*r* = −0.25; *p* = 0.013), risk factors (*r* = −0.36; *p* < 0.001), and preventive aspects (*r* = −0.24; *p* = 0.019). On the other hand, compliers demonstrated a significant higher knowledge in the prevalence of the disorder (*p* = 0.016). In particular, when inquired about the risk factors affecting the occurrence of peri‐implantitis, diabetes and implant and prosthesis characteristics were commonly unknown. On the other side, they admitted the significance of poor oral hygiene, poor compliance, and smoking as contributors to peri‐implantitis (Figure 3). Compliers and non‐compliers also demonstrated significant differences regarding their knowledge of the risks of not attending supportive therapy sessions (*p* = 0.041).

### 3.2 Knowledge on treatment

**FIGURE 4:**
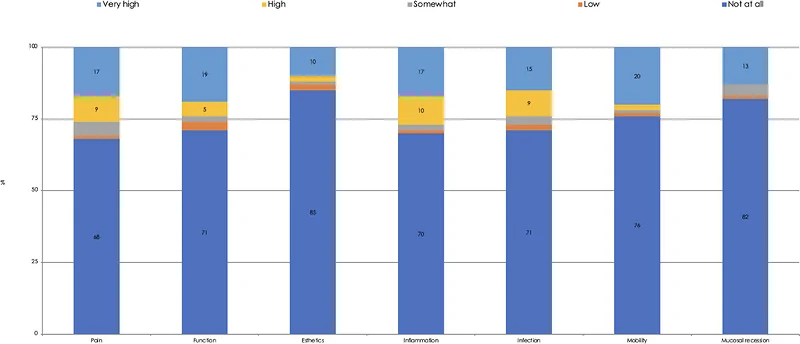
Overall concern immediately following peri‐implantitis treatment.

Patients disclosed general unknown concerning multiple factors that could be implied on disease recurrence following treatment. Notably, diabetes, implant and prosthesis characteristics were the factors that were not perceived by patients as likely to increase likelihood of disease recurrence, while poor plaque control, smoking, and lack of compliance were recognized to be associated with disease recurrence. Again, compliers recognized the deleterious impact of not attending supportive therapy sessions when compared to non‐compliers (*p* = 0.004). Similarly, compliers regarded the influence of implant surface characteristics as a factor in increasing odds of disease recurrence (*p* = 0.010). Interestingly, the vast majority of patients (70%–85%) were not concerned about pain, esthetic outcome, inflammation, or other sequelae related to therapy immediately after delivering the treatment (Figure 4). It is relevant to note that patients supported at the Department of Periodontology at UIC Barcelona displayed more pain (*p* = 0.02) and concern about the esthetic outcome (*p* = 0.001) in contrast to the enrollment of maintenance of the dental school and later on maintained in our department. Compliers were, likewise, more concerned about exposure of the implant and mucosal recession following treatment (*p* = 0.02) when compared to non‐compliers. Similar outcomes were achieved when inquired about their perspective at the re‐evaluation. In fact, concern regarding pain, esthetic outcome, inflammation or other sequelae related to therapy following re‐evaluation dropped further. Compliers demonstrated statistical significance in identifying inflammation (*p* = 0.02), persistence of infection (*p* = 0–009), implant mobility (*p* = 0.010), and other peri‐implant problems such as exposure or recession (*p* = 0.02) when compared to non‐compliers following re‐evaluation.

### 3.3 Perception of peri‐implantitis and its treatment

**FIGURE 5:**
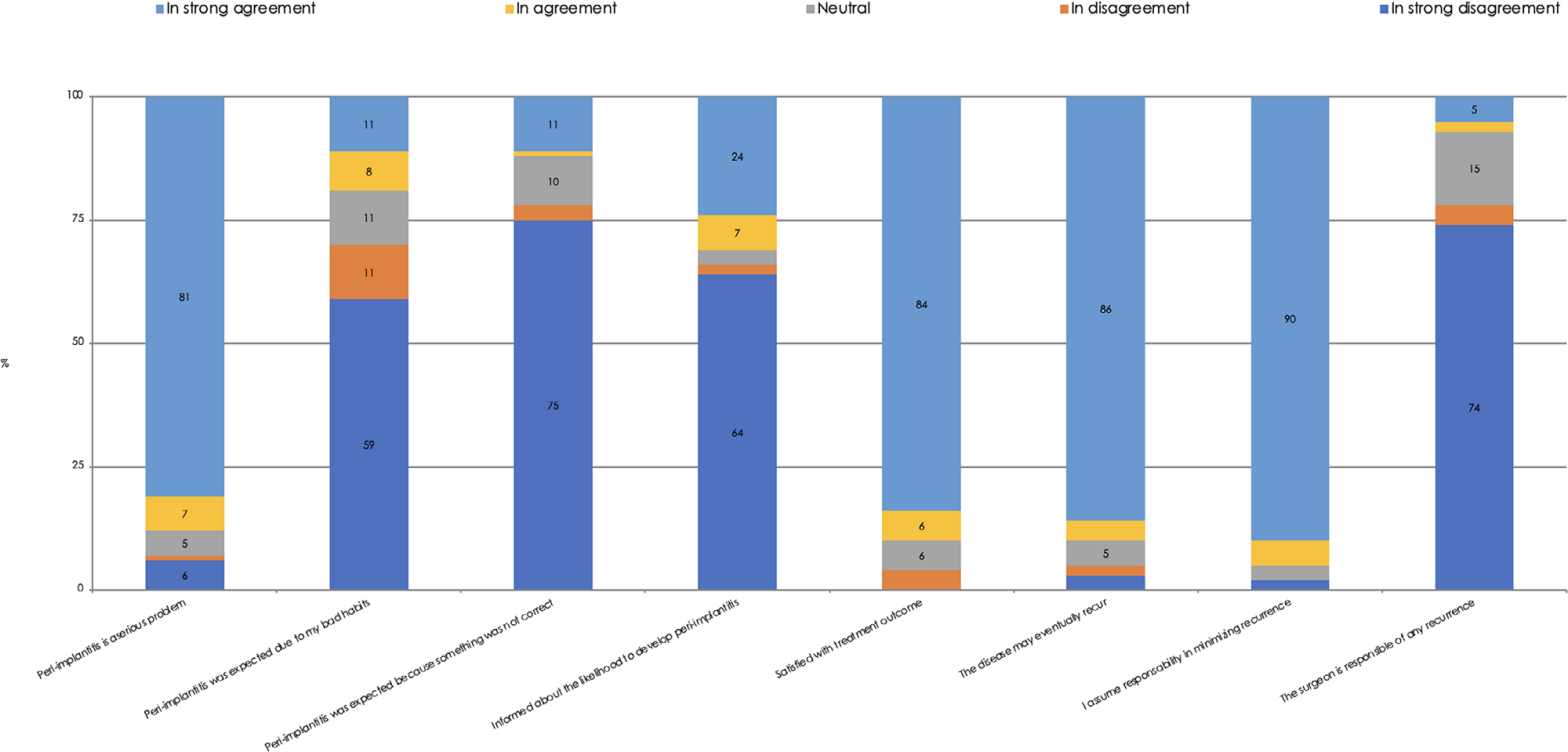
Level of agreement in patients´ perception following the treatment of peri‐implantitis.

**FIGURE 6:**
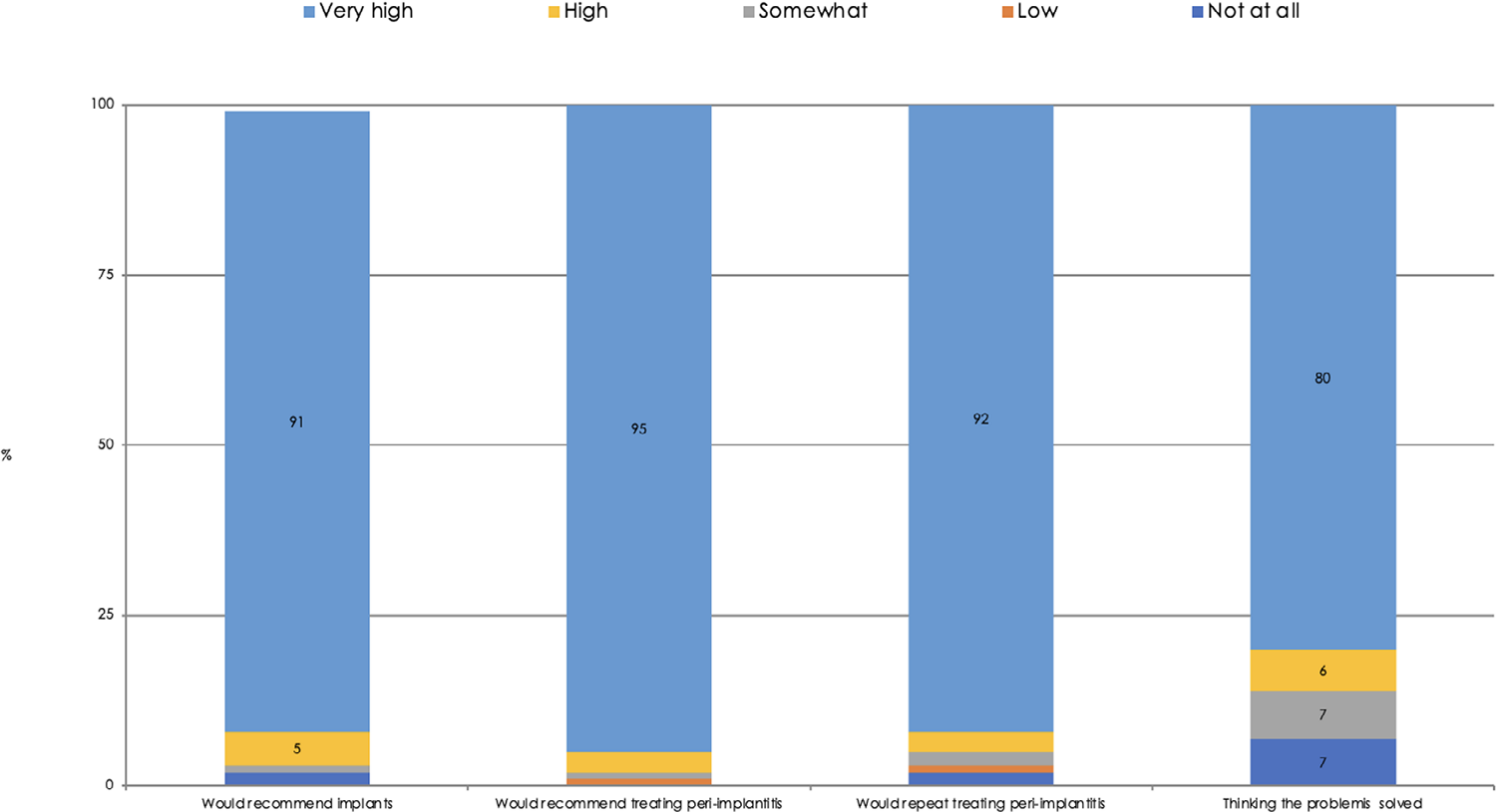
Overall patients´ satisfaction following the treatment of peri‐implantitis.

More than 90% of patients, despite the diagnosis of peri‐implantitis, would recommend implant therapy, treatment of peri‐implantitis, and would accept to repeat the treatment that they were recommended. Overall, 80% of patients thought that their problem was completely solved by the therapeutic intervention for peri‐implantitis. Interestingly, 7% showed remarkable disagreement about this item. Patients acknowledged peri‐implantitis as an infectious disease. However, 64% of individuals were dissatisfied with the lack of information about the incidence of peri‐implantitis prior to implant therapy. These patients did not expect peri‐implantitis to be linked to bad habits or to be blamed for something that was not correctly executed, displaying, therefore, a low level of knowledge (Figure 5). Interestingly, patients supported following peri‐implantitis treatment at the Department of Periodontology at UIC Barcelona were significantly more informed about the incidence of peri‐implantitis (*p* = 0.005) compared to those receiving therapy elsewhere prior to implant therapy. Moreover, they were overall satisfied with the treatment outcome and they were aware that disease may eventually recur assuming their role in minimizing disease recurrence applying healthy habits (Figure 6).

### 3.4 Perception following treatment outcome

Overall, 63% of the patients successfully responded to therapy displaying arrest in progressive bone loss, 26% further demonstrated progressive bone loss (≥ 1 mm), and 11% were subjected to implant removal. Interestingly, when correlating overall patients´ satisfaction according to their treatment outcome, only a marginal, but not statistically significant, association was elucidated on the item “thinking that the problem was solved” (*r* = 0.17; *p* = 0.09). Alike, patients´ perception of peri‐implantitis was not altered according to their therapeutic outcome. Hence, treatment outcome did not have an effect on patients´ perception and satisfaction (Supplementary Table 2).

## 4 DISCUSSION

Dental implants replace missing teeth in ∼ 100 million people in the world, nearly 20% of implants are prone to develop peri‐implantitis. These data are in fact alarming; however, the high proportion of patients with inaccurate perceptions and unrealistic expectations of implant therapy makes these data further disturbing.^5^, ^6^ Ignorance concerning the likelihood to experience disease, the nature of the problem, and factors associated with the occurrence of peri‐implantitis have been featured.^10^ In fact, ∼70% of patients disclose that peri‐implantitis is a rare complication and that implants are life‐lasting devices.^8^ This study performed in an academic setting aimed at elucidating the knowledge and perception on factors associated with the occurrence of peri‐implantitis and its treatment in patients that were subjected to therapy. In agreement with previous findings,^5^, ^7^, ^8^, ^10^ the overall level of knowledge about peri‐implantitis is moderately low, where ∼ 60% of the patients surveyed reported null knowledge about diagnosis, prevalence, or risk factors associated with disease occurrence despite have being subjected to treatment of this disease. However, in general, patients acknowledged that poor plaque control, smoking, and not attending regular check‐ups might be involved in increasing disease onset. In fact, compliers to supportive care yielded a greater level of concern and knowledge regarding aspects related to peri‐implantitis and its recurrence. Interestingly, patients were overall satisfied immediately (following therapy), at early (re‐evaluation), and at late stages (during follow‐up) with therapeutic outcomes of peri‐implantitis. Patients accept the possibility of disease recurrence and their role in minimizing it. In summary, these findings enlighten few key aspects to be translated to the clinical basis: (1) in light of the low level of knowledge, patients must be meticulously informed about the odds to develop implant‐related complications and the factors associated with an increase likelihood, and (2) peri‐implantitis therapy does, in general, satisfy the patients and do not deteriorate their quality of life. However, patients do not seem to be homogeneously educated about risk that may enlighten the odds for disease recurrence. These findings, anyway, must be interpreted with cautiousness, given that the cohort of patients was recruited in an academic institution where the costs associated with these treatments and their expectations are, in general, lower to private practices and given that a large sample of patients were only subjected to non‐surgical/flapless submucosal instrumentation therapy. Surgical interventions, instead, might have led to lower level of esthetic satisfaction due to mucosal recession, especially resective approaches, and a higher level of post‐operative disconfort.^14^ Despite of that, the type of intervention did not show significance in any of the items explored; hence, similar outcomes may also expected in patients subjected to surgical approaches.

Patients´ experiences of implant therapy, peri‐implantitis, and its treatment were explored in a qualitative interview study. It was noticed that implant therapy improved numerous aspects of the patients’ quality of life. Alike, peri‐implantitis did not expose the patients to major discomfort, being local anesthesia the worst part of therapy.^7^ In fact, previously, altered expectations on implant therapy in patients referred for treatment of peri‐implantitis were elucidated. It was demonstrated that unrealistic high expectations often lead to frustration when biologic complications are diagnosed.^19^ Data demonstrated that 74% of patients were not informed about the possible occurrence of peri‐implantitis in the long‐term.^10^ Likewise, roughly the same number of patients had the erroneous belief that implants are life lasting devices.^10^ The low level of knowledge and awareness on disease prevalence, risk factors, and sequalae seem to be even across the globe.^10^, ^19^, ^20^, ^21^ Our study further supported the little patient‐centered understanding of factors associated with peri‐implantitis. This is not surprising since, even among periodontists, there seems to be inconsistencies.^16^ However, when disease occurs, most of patients acknowledge that patient‐related deleterious habits such as poor plaque control, lack of compliance to supportive care, or smoking may contribute to disease initiation and progression. This highlights that, despite education enhancement to provide patients realistic expectations is key, it is also relevant to meticulously assess patients´ risk profile to engage healthy habits that can be conducive to long‐term success.

Long‐term treatment outcomes of peri‐implantitis demonstrated effectiveness.^22^ Mean disease resolution rate based on composite case definition was 58.6%; mean progressive bone loss arrest (> 1 mm) at the latest follow‐up following therapy was 69.6%.^22^ In agreement, outcomes from this retrospective study yielded 63% of bone loss arrest. Regrettably, clinical data during baseline examination were not consistently reported. Thereupon, disease resolution could only be evaluated with the latest radiographic and clinical follow‐up data. Anyway, contrary to our hypothesis, treatment outcomes (i.e., progressive bone loss, arrest of progressive bone loss, or implant removal) did not impact upon patients´ perception or satisfaction. This may indicate the lack of transparency from the operators and examiners during follow‐up concerning the actual prognosis according to the re‐evaluation or the unrealistic over‐expectations from the patients concerning their treatment outcome.

Patient‐centered outcomes following the treatment of peri‐implantitis have been seldomly reported. A prospective clinical study in a limited number of individuals assessed quality of life following the surgical therapy of peri‐implantitis using a visual analog scale to report post‐operative pain on a daily basis and completing the OHIP‐14sp questionnaire.^15^ Interestingly, low to moderate deterioration was perceived in oral health following therapy of peri‐implantitis. In fact, 80% of patients were satisfied with treatment, and 36% would not repeat the treatment.^15^ In agreement, data from the present study demonstrate that 83% of patients were satisfied with the treatment outcome. On the other hand, 92% of the patients enrolled in the present study would recommend the treatment whenever needed. This notable difference might be attributable to the time of assessment. While we tested this question in the following healing, the former study inquired about it during healing. Hence, moderate pain experienced immediately after the surgical intervention may explain this discrepancy. In addition, most of the cases included in our study were managed by non‐surgical/flapless means, and it may have further contributed to this outcome.

It is important to highlight shortcomings of this study. First, clinical records were not collected because variability among examiners could have affected the accuracy of defining disease resolution. Instead, the authors considered it more appropriate to set arrest of progressive bone loss beyond the standard of error of measurement as an endpoint.^18^ In this sense, periapical radiographs were obtained using the long‐cone paralleling technique; however, they were not standardized. In addition, the high level of patient‐reported satisfaction with the therapy may be partly attributed to the fact that the majority of interventions involved non‐surgical measures. Therefore, caution is advised when interpreting this finding. It is also important to consider that the reluctance of many patients to undergo surgical interventions may be related to financial constraints. Furthermore, it should be noted that patients did not receive the same level of information, as implant therapy was not consistently delivered by the same operators. Consequently, the lack of control over the information provided to patients, as well as over behavioral habits prior to implant placement, may have influenced the outcomes. Future studies should aim to standardize the information delivered in order to better assess patients’ level of awareness during follow‐up. Additionally, it should be kept in mind that the method used to determine patients’ responses was based on previously published reports.^10^, ^16^, ^23^ However, it may be debatable whether this represents the most accurate method for collecting responses. Overall, the findings of this study highlight the importance of comprehensive communication before subjecting a candidate to implant therapy, as well as before selecting a specific approach to arrest disease progression when peri‐implantitis is diagnosed. In this context, appropriate patient selection is of paramount importance to increase the likelihood of successful outcomes.

## 5 CONCLUSION

Patient‐reported information on factors related to peri‐implantitis occurrence and recurrence is generally unsatisfactory. Nearly 60% of patients are unaware of factors related to peri‐implantitis occurrence. However, peri‐implantitis therapy does not seem to negatively interfere in the patients´ quality of life or satisfaction, despite the treatment outcomes, in patients subjected to treatment. In this sense, patients accept the likelihood of having disease recurrence.

## AUTHOR CONTRIBUTIONS

All the authors conceived the concept. M.S.R. and M.I. performed the screening. P.K., J.N., C.V. supervised the writing. A.M. conceived the concept. M.S.R. and I.G. performed the screening. P.K., J.N., C.V. supervised the writing.

## CONFLICT OF INTEREST STATEMENT

The authors declare no conflicts of interest.

## FUNDING INFORMATION

The authors received no specific funding for this work.

## Supporting information



Supporting information

## Data Availability

The data that support the findings of this study are available from the corresponding author upon reasonable request.
