# Supplementary material for: Understanding peri‐implantitis occurrence and recurrence following treatment: A patient‐centered study
Source: J Periodontol. 2026 Apr 28;97(9):1980–9. doi: 10.1002/jper.70133 (PMC13569071; doi:10.1002/jper.70133)
Supplement: Supplementary file 1 [file JPER-97-1980-s001.docx]

**Supplementary table 1.** Survey-based questionnaire

**Universitat Internacional de Catalunya – School of Dentistry**

**Survey concerning understanding and perception of peri-implantitis and its treatment**

**Thank you very much for participating in this study. Let us assure you that your responses are completely anonymous. All results will be reported on an aggregate level.**

**Let us start with some questions about your background:**

**1.	Are you male or female?	Male** 🞐	**Female** 🞐

**2.	How old are you?	I am ______ years old.**

**3.	How many dental implants do you have? _____**

**4. Were they installed at the school? _____ If yes, which department? _________**

**5. How long ago were the implants installed? ______ years**

**6. Are you still attending for regular check-ups? ______ if yes, with what periodicity? ___x/year**

**7. When was your implant infection (peri-implantitis) diagnosed? ______ years**

**8. Were you alarmed by any symptom/sign? ________ if yes, answer item 9. If no, answer item 10**

**9	Sign/symptom: Bleeding** 🞐	**Plus** 🞐 **Pain** 🞐	**Bad breath** 🞐 **Swelling** 🞐

**10.	Who did you notice about the infection? Dentist** 🞐	**Periodontist** 🞐 **Dental hygienist** 🞐 **other**🞐

**11.	How many implants were infected with peri-implantitis? ______**

**12. How well were you educated about	Not at all	Very well**

**a.	how to diagnose peri-implantitis?	1	2	3	4	5**

**b.	the risk factors of peri-implantitis?	1	2	3	4	5**

**c.	preventing peri-implantitis?	1	2	3	4	5**

**d.	prevalence of peri-implantitis?	1	2	3	4	5**

**13.	How much do the following factors put a patient at risk for developing peri-implantitis?**

**Not at all	Very	Don’t know**

a. **Poor oral hygiene	1	2	3	4	5**	🞐
b. **History of periodontitis	1	2	3	4	5**	🞐
c. **Smoking	1	2	3	4	5**	🞐
d. **Diabetes mellitus	1	2	3	4	5**	🞐
e. **Genetic predisposition	1	2	3	4	5**	🞐
f. **Implant surface	1	2	3	4	5**	🞐
g. **Occlusion	1	2	3	4	5**	🞐
h. **Presence of keratinized tissue	1	2	3	4	5**	🞐
i. **Not attending to checks at least 2x/year 1	2	3	4	5**	🞐
j. **Surgeon skills	1	2	3	4	5**	🞐
k. **Type of implant	1	2	3	4	5**	🞐
l. **Type of prosthesis	1	2	3	4	5**	🞐

**If other, please explain: ____________**

**14. In how many implants did you receive treatment for the infection? ______**

**15. How long ago was the intervention? ______ years**

**16. What type of treatment did you receive? Non-surgical** 🞐 **Surgical** 🞐

**16. Did you receive any of the following as part of treatment? Bone graft** 🞐 **Membrane** 🞐 **Soft tissue graft** 🞐

**17. Was laser used for the treatment? Yes** 🞐 **No** 🞐

**18. Were you delivered antibiotics after treatment? Yes** 🞐 **No** 🞐 **If yes, for how long? _____**

**19. Were you delivered pain killers after treatment? Yes** 🞐 **No** 🞐 **If yes, for how long? _____**

**20. Were you delivered mouth rinse after treatment? Yes** 🞐 **No** 🞐 **If yes, for how long? _____**

**21. Did the implant survive after treatment? Yes** 🞐 **No** 🞐 **If no, how did it last thereafter? _____**

**22. Were you noticed about the success rate? Yes** 🞐 **No** 🞐 **If yes, how much you expect was the rate? _____**

**23.	How much do the following factors put a patient at risk for developing recurrence of peri-implantitis?**

**Not at all	Very	Don’t know**

m. **Poor oral hygiene	1	2	3	4	5**	🞐
n. **History of periodontitis	1	2	3	4	5**	🞐
o. **Smoking	1	2	3	4	5**	🞐
p. **Diabetes mellitus	1	2	3	4	5**	🞐
q. **Genetic predisposition	1	2	3	4	5**	🞐
r. **Implant surface	1	2	3	4	5**	🞐
s. **Occlusion	1	2	3	4	5**	🞐
t. **Presence of keratinized tissue	1	2	3	4	5**	🞐
u. **Not attending to checks at least 2x/year 1	2	3	4	5**	🞐
v. **Surgeon skills	1	2	3	4	5**	🞐
w. **Type of implant	1	2	3	4	5**	🞐
x. **Type of prosthesis	1	2	3	4	5**	🞐

**If other, please explain: ____________**

**24.	Immediately after the treatment, how concerned were you in terms of ….?**

**Not at all	Very**

**a.	Pain	1	2	3	4	5**

**b.	Function	1	2	3	4	5**

**c.	Esthetics	1	2	3	4	5**

**d.	Inflammation	1	2	3	4	5**

e. **Persistence of infection	1	2	3	4	5**
f. **Implant mobility	1	2	3	4	5**
g. **Implant exposure and gum recession	1	2	3	4	5**
h. **Other	1	2	3	4	5**

**25.	After re-evaluation, how concerned were you in terms of ….?**

**Not at all	Very**

**a.	Pain	1	2	3	4	5**

**b.	Function	1	2	3	4	5**

**c.	Esthetics	1	2	3	4	5**

**d.	Inflammation	1	2	3	4	5**

i. **Persistence of infection	1	2	3	4	5**
j. **Implant mobility	1	2	3	4	5**
k. **Implant exposure and gum recession	1	2	3	4	5**
l. **Other	1	2	3	4	5**

**26. How much would you recommend dental implants given the complications associated?**

**Not at all	Very**

**1	2	3	4	5**

**27. How much would you recommend treating peri-implantitis?**

**Not at all	Very**

**1	2	3	4	5**

**28. How much would you repeat treating peri-implantitis?**

**Not at all	Very**

**1	2	3	4	5**

**29. 27. Do you think the problem has been solved?**

**Not at all	Very**

**1	2	3	4	5**

**30.	On a scale from 1 = disagree strongly to 5 = agree strongly, how much do you disagree/agree with the following statements?	Disagree	Agree**

**Strongly	Strongly**

**a. I consider peri-implantitis a serious problem.	1	2	3	4	5**

**b. I was expecting peri-implantitis given my bad habits	1	2	3	4	5**

**c. I was expecting peri-implantitis since I knew from**

**the beginning that something wasn’t being correctly	1	2	3	4	5**

**d. I was informed about the likelihood to develop it. 1	2	3	4	5**

**e. I am satisfied with the treatment outcome	1	2	3	4	5**

**f. I understand that the disease may eventually recur	1	2	3	4	5**

**g. I assume my responsibility in minimizing recurrence	1	2	3	4	5**

**h. I consider the surgeon responsible in any recurrence	1	2	3	4	5**

**Please share with us any other thoughts/considerations you have about peri-implantitis:**

**Thank you very much for participating in this study. If you have any questions about this survey or are interested in the results of this study, please contact Dr. Alberto Monje at** amonjec@umich.edu

**Supplementary table 2.** Results of Kruskal-Wallis´ test and Pearson´s r correlation between patients’ perception and treatment outcome.

|  | **Less successful therapeutic outcome** | **Weighted grade** |
| --- | --- | --- |
| Peri-implantitis is a serious problem | 0.143 | r= -0.10; p=0.311 |
| Expecting peri-implantitis due to my bad habits | 0.515 | r= 0.07; p=0.519 |
| Expecting peri-implantitis due to something no correctly performed | 0.926 | r= -0.04; p=0.732 |
| Informed about likelihood to develop | 0.308 | r= -0.13; p=0.185 |
| Satisfied with treatment outcome | 0.243 | r= 0.13; p=0.216 |
| The disease may eventually recur | 0.556 | r= 0.06; p=0.559 |
| I assume responsibility in minimizing its recurrence | 0.373 | r= 0.07; p=0.493 |
| The surgeon is responsible for recurrence | 0.969 | r= 0.05; p=0.619 |
